# Conference Report: NORTH AMERICAN INTEGRATED SERVICES DIGITAL NETWORK (ISDN) USERS’ FORUM (NIUF) Gaithersburg, MD June 22–25, 1993

**DOI:** 10.6028/jres.098.041

**Published:** 1993

**Authors:** Elizabeth B. Lennon

**Affiliations:** Computer Systems Laboratory, National Institute of Standards and Technology, Gaithersburg, MD 20899-0001

## 1. Introduction

The Computer Systems Laboratory (CSL), National Institute of Standards and Technology, hosted the eighteenth North American ISDN Users’ Forum (NIUF) at its Gaithersburg, Maryland, site on June 22–25, 1993. About 230 users, implementors, and service providers of ISDN technology attended the meeting. CSL collaborated with industry in 1988 to establish the NIUF to ensure that emerging ISDN applications meet the needs of users. A Cooperative Research and Development Agreement (CRDA) with industry was established in 1991 to govern the management of the forum; as of June 1993, the CRDA has 36 signatories from industry and academia. CSL serves as chair of the forum and hosts the NIUF Secretariat. NIUF membership is open to all interested users, product providers, and service providers; meetings are held three times a year at various locations throughout North America.

## 2. The Development of ISDN Standards

International standards for ISDN support worldwide communications for the exchange of voice, data, and image information among users, independent of any manufacturer, service provider, or implementation technology. ISDN standards are developed by the International Telecommunication Union-Telecommunication Standardization Sector (ITU-TS) (formerly the International Telephone and Telegraph Consultative Committee [CCITT]) and, for North America in particular, by the Exchange Carriers Standards Association (ECSA) accredited standards committee, T1, under the umbrella of the American National Standards Institute (ANSI).

ISDN standards provide a broad variety of options and parameters to meet many potential needs and applications. To ensure interoperability and terminal portability within the ISDN network and its attendant equipment, a uniform subset of options and parameters must be selected for implementation. Each application usually requires only a subset of total functionality available in the standards; for ISDN products and services to work together in a multi-vendor environment, common sets of options must be selected.

To cope with this proliferation of choices and to provide interoperable products and services which will meet the needs of users, the standards specification process has been augmented to develop application profiles, implementation agreements, and conformance criteria. The NIUF addresses all of these areas.

## 3. NIUF Objectives and Structure

The NIUF seeks to achieve three principal goals:

- To promote an ISDN forum committed to providing users the opportunity to influence developing ISDN technology to reflect their needs;
- To identify ISDN applications, develop implementation requirements, and facilitate their timely, harmonized, and interoperable introduction; and
- To solicit user, product provider, and service provider participation in the process.

The actual work of the NIUF is accomplished in two workshops: the ISDN User’s Workshop (IUW) and the ISDN Implementor’s Workshop (IIW). The IUW produces application requirements which describe potential applications of ISDN and the features which may be needed. The IIW develops application profiles, implementation agreements, and conformance criteria which provide the detailed technical decisions necessary to implement an application requirement in an interoperable manner. The NIUF Executive Steering Committee coordinates the activities of the two workshops.

## 4. NIUF Achievements

Since its inception in 1988, the NIUF has achieved the following:

- 141 active applications for development of application profiles have been accepted;
- application profiles have been completed for 14 applications;
- 15 implementation agreements have been completed; and
- 8 conformance tests have been completed.

CSL established the NIST Special Publication 823 series, Integrated Service Digital Network Technology Publications, to publish the approved implementation agreements, conformance tests, and other NIUF documents. Copies of these documents are available for sale by the U.S. Government Printing Office, (202) 783-3238 or the National Technical Information Service, (703) 487-4650.

## 5. Highlights of June 1993 NIUF

Tutorials presented at the June meeting included an overview of the NIUF for new users and new implementors; migration to broadband ISDN; basic ISDN; applications software interface; and national ISDN user applications.

Highlights from the Executive Steering Committee Standing Groups included a presentation by Pat Donovan, Bell Atlantic, on the provisional definition of National ISDN-3. The presentation was part of the National ISDN Planning Process that is an ongoing part of NIUF activities. The panel discussion following the talk featured representatives from AT&T, NTI, Siemens Stromberg-Carlson, Ericsson, and Ameritech.

The IUW held its first General User Meeting in order to cover topics of interest to a wide range of users and implementors. A resounding success in terms of attendance and interest generated by the topics, the meeting focused on three main topics: the current state of ISDN tariffs in the United States and Canada; the status of the Tennessee Public Utilities Commission ISDN field trials; and a panel discussion on the impact of the Clean Air Act Amendment on state and local governments and the use of telecommuting as a potential solution.

IUW Working Group highlights included the following: the Broadband ISDN Working Group presented tutorials on Switched Multimegabit Data Service (SMDS) and the North Carolina Broadband Network; the Enterprise Network Data Interconnectivity Family (ENDIF) discussed the need for interoperability among different vendors for ISDN applications involving access to LANs and between LANs, and proposed a demonstration for the October meeting; the Government Services Industry Group (GSIG) identified three high-profile items for the GSIG to address through the forum: ISDN and GOSIP compatibility, the definition of managed objects for secure network management, and secure video conferencing; the Private Industries Group decided to try to develop an initial Quality Index figure to measure the availability and usability of ISDN as it applies to the companies represented, and discussed packet data services over ISDN and the lack of well-defined service parameters among the various exchange carriers; the Call Management Family continued to advance the Telecommuting Application Profile, with the goal of presenting a stable draft at the October NIUF; and the Messaging and Answering Family heard a presentation by Jim Rothweiler, Bellcore, on a survey assessing five new features that will support the voice messaging industry in providing service to the consumer market.

The IIW approved three documents and declared six documents as working group stable (see plenary highlights below). Glenn Ehley, IIW Chair, proposed an increased participation drive. One recommendation was the use of graduate students to assist with the application analysis process. Also identified was the need for a Broadband Application Profile Team.

IIW Working Groups reported the following activities: ISDN CPE and Software Working Group (ICSW) heard presentations on the implementation schedules for National ISDN-2. Ameritech sponsored a session on the Primary Rate ISDN switched fractional services that will become available with National ISDN-2. The ICSW Basic Rate Subcommittee hopes to implement a simplified ISDN ordering procedure by year’s end. The ICSW PBX Subcommittee began an effort to facilitate the standardization of the ISDN-based Q.SIG protocols for ISDN PBX interworking. The Application Analysis Technical Working Group helped to advance work on “Interactive Simulation.” The forum discussed the initial draft of the second edition of “A Catalog of National ISDN Solutions for Selected NIUF Applications.” Proposed publication date is February 1994. The Call Management Profile Team met in three sessions with other groups to discuss the Telecommuting Application Profile. The CPE Compatibilities and Capabilities Profile Team will develop an implementation plan to attract graduate students. The ISDN Conformance Testing Technical Working Groups reported progress in their individual arenas. The Signaling/Supplementary Services (SSWG) Technical Working Group discussed Class II, BRI Trunking, and received support from users and implementors. Finally, the Video/Audio Conferencing Profile Team gave a second review to the Video Conference Application Profile document, which was declared working group stable once two open issues have been resolved.

At the NIUF Banquet, Matt Thomson, Northern Telecom, received a certificate of recognition for his leadership in the NIUF and his work on TRIP ’92. InfoWorld magazine recognized the NIUF and Corporation for Open Systems (COS) with the Publisher’s Industry Milestone Award for their efforts in the success of TRIP ’92.

The closing plenary approved five new documents, including “A Generic Model for ISDN Cost Analysis.” Also approved were Working Group Charters for Mass Market Industries; CPE Compatibilities and Capabilities Family; and Issues Family. Seven documents were announced to be working group stable and five new applications were submitted to the IUW.

## 6. For More Information

For more information about the NIUF and its publications or to obtain conference proceedings, contact the NIUF Secretariat: Dawn Hoffman, Computer Systems Laboratory, National Institute of Standards and Technology, Building 223, Room B364, Gaithersburg, MD 20899-0001; telephone (301) 975-2937 or fax (301) 926-9675.

